# Occupational protection behavior and its influencing factors of newly recruited nurses

**DOI:** 10.1186/s12909-023-04780-6

**Published:** 2023-10-25

**Authors:** Yang Xu, Wen-jie Liu, Xia Wang, Qian-mei Yang

**Affiliations:** 1 Department of Neurosurgery, West China Hospital, Sichuan University, Chengdu, Sichuan PR China; 2 School of Nursing, West China Hospital of Sichuan University, Chengdu, Sichuan PR China

**Keywords:** Occupational protective behavior, Work attitude, Influencing factors, New recruits, Nurse, Needlestick and sharps injuries

## Abstract

**Aim:**

Aim The objective of this study was to understand the occupational protective behaviors of newly recruited nurses and explore the influencing factors.

**Methods:**

A convenience sampling method was used to select newly recruited nurses in our hospital from July 2018 to November 2019. The survey was conducted using the general information questionnaire, work attitude scale (Wa), and occupational protective behavior scale.

**Results:**

The total score of occupational protective behaviors of 150 newly enrolled nurses was 18.94 ± 3.59. There was a significant negative correlation between work attitude score and occupational protective behaviors (r = -0.324, *p* < 0.001). Multiple linear regression analysis showed that gender, previous participation in nursing skill-based competitions, experience of needlestick injuries before recruit, work attitude score, average daily sleep time (*p* < 0.05) were independent factors influencing occupational protective behaviors.

**Conclusions:**

The overall occupational protective awareness of newly enrolled nurses is relatively weak and needs to be further improved. The group’s ability to improve occupational protective behaviors may be positively impacted through increased adaptability, improved sleep, active participation in nursing skill-based competitions, strengthening guidance and education on occupational protection.

**Supplementary Information:**

The online version contains supplementary material available at 10.1186/s12909-023-04780-6.

## Introduction

Needlestick and sharps injuries are skin injuries caused by objects contaminated with potentially contagious material [1]. In addition, skin and mucous membranes can be exposed to potentially infectious body fluids through splashes. Needlestick and sharps injuries are the most common work-related accidents among health care workers (HCW) [2]. The incidence of self-inflicted injuries has been reported in the literature as 1.4–9.5/100 HCW/year [3]. Needlestick and sharps injuries carry a risk of occupational infection and more than 60 different pathogens have been described [4], with transmission of hepatitis B virus (HBV), hepatitis C virus (HCV), and human immunodeficiency virus (HIV) playing a dominant role [3]. In the event of occupational exposures such as needlestick injuries and healthcare-acquired infections [5, 6], this can lead to high levels of psychological consequences such as stress, anxiety and depression on the one hand, and an increased healthcare burden on the other hand [7, 8]. Therefore, it is of great importance to take a series of measures to reduce the occurrence of occupational exposures such as needlestick and sharps injuries.

Within the HCW, the incidence of occupational exposures in nurses is close to 20% [1], which may be related to inadequate management practices and insufficient educational programs [1, 6, 9, 10]. With increased awareness of occupational exposures, a range of protective equipment such as gloves, aprons and/or gowns, and eye protection, is an important aspect of infection prevention and control for all HCW, including nurses [11, 12]. However, optimal use of protective equipment is often difficult and HCW may change the delivery of care because of protective equipment [11]. Studies have found that new practitioners, such as residents or interns, are the most vulnerable and susceptible to occupational exposures among HCW [13]. In clinical practice, we have found that newly recruited nurses have a higher incidence of occupational exposures than experienced nurses. The newly recruited group not only try to adapt to the working environment, but also to the requirements of the job as soon as possible. Whether this will have an adverse effect on their occupational protective behaviors remains to be studied.

The above reports revealed a status quo that advanced protective equipment alone cannot ensure occupational safety of these newly recruited groups. Lack of experience and non-compliance with precautionary measures are also important causes of their occupational exposures. Therefore, it is important to gain an in-depth understanding of the current status of occupational protective behaviors in these groups, and to provide special treatment for potential risk factors, such as education and practice training, which will in turn reduce the occurrence of occupational exposures. At present, there are few studies on the occupational protective behaviors of newly recruited nurses. Therefore, this study is intended to investigate the current situation of occupational protective behaviors and possible risk factors of newly recruited nurses in order to provide a scientific basis for guiding hospital administrators to develop better occupational protective measures and improve the management and education of nurses.

## Materials and methods

### Subjects

A convenience sampling was used to select newly recruited nurses in our hospital from July 2018 to November 2019 as the study population for the survey. To be eligible for the study, participants needed to meet the following criteria: (1) registered nurses; (2) willing to participate in this study with informed consent; (3) new recruit ≤ 1 year. Exclusion criteria were as follows: (1) more than 1 year of nursing experience; (2) previous medical malpractice during work; (3) unregular employees (who work part-time). Exclusion criteria also included alcohol or drug addiction, psychiatric disorders, concomitant chronic diseases which can influence the psychiatric status. The sample size of the cross-sectional study was calculated using 10 ~ 20 times the number of dimensions, and 15 times of the median value was taken to obtain: (8 + 1 + 1)*15 = 150 (cases) [14]. According to the data provided by the Department of Infection and Control of our hospital, the incidence of needlestick and sharps injuries among newly recruited nurses between July 2018 to November 2019 was 13.3% (20/150), which was significantly higher than the incidence of occupational exposures among nurses with 2 years of working experience (5.8%). Before the study began, we had carefully consulted the Ethics Committee and Institutional Review Board of West China Hospital. They suggested that this study did not involve special interventions for the participants, and we should conduct this study in compliance with the Helsinki Declaration and inform the participants fully of the purpose of the study. So, all data was fully anonymised at source with researchers. The need for Informed Consent was waived by the Ethics Committee and Institutional Review Board of West China Hospital due to the retrospective nature of the study.

### Questionnaires

#### General information questionnaire

The general information questionnaire was self-designed and included gender, age, education background, marital status, previous participation in nursing skill-based competitions, standardized training before recruit, experience of needlestick injuries before recruit, and average daily sleep time.

#### Work attitude scale (wa)

The scale was first developed by Tyollaska in 1953. It contains 37 items, of which 29 items are counted as “yes” and 8 items are counted as “no”, and the score ranges from 0 to 37 points (Supplementary material 1). The higher the score, the worse the individual’s work adaptability and motivation [15]. The Cronbach’s α coefficient for this study was 0.793.

#### Occupational protective behavior scale

**Fig. 1:**
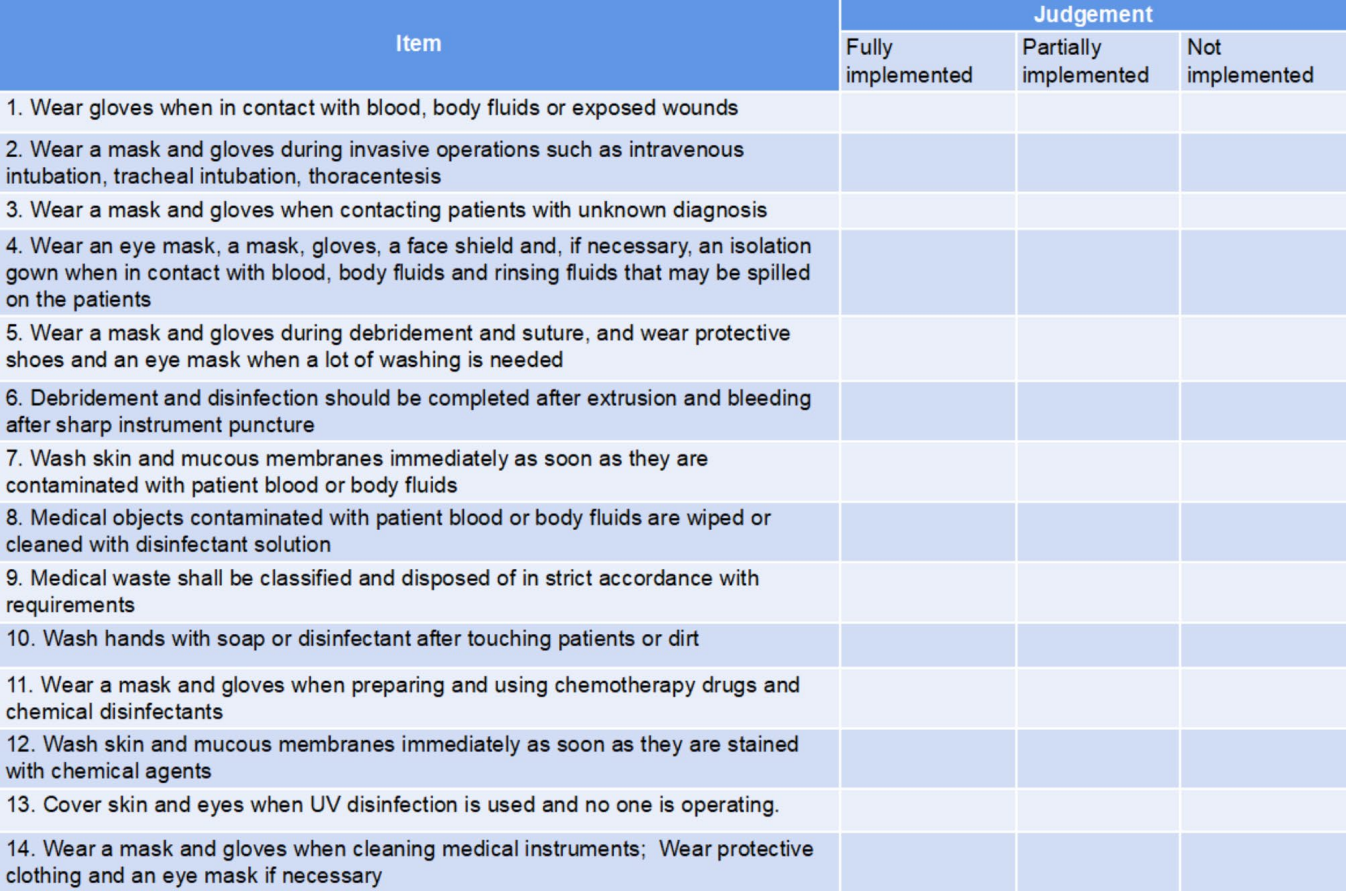
Occupational protective behavior scale

The scale was designed by the researchers on the basis of extensive literature review [16], and five nursing experts from our hospital were invited to review and modify it (Supplementary material 2). The content validity index of the scale was 0.857. Thirty newly recruited nurses who met the requirements were selected for the preliminary experiment, and the Cronbach’s α coefficient was 0.804, indicating that the scale had good reliability and validity. There were 14 items in total, and each item was graded at 3 levels: “fully implemented” =2 points, “partially implemented” =1 point, and “not implemented” =0 point (Fig. 1). The score ranged from 0 to 28 points. The higher the score, the better the occupational protective behaviors. Cronbach’s α coefficient of this study was 0.837.

### Investigation procedure

Firstly, the investigators were trained uniformly and these investigators administered the questionnaires to the newly enrolled nurses in strict accordance with the survey criteria and guidelines. Before the start of the survey, informed consent should be obtained from the nurses enrolled in this study, and they should voluntarily join this study, and they should be informed of the precautions during the completion of the questionnaire. During the survey, experimental bias should be strictly controlled to avoid contamination among nurses, and the questionnaires should be completed independently. After the completion of the survey, all questionnaires should be retrieved in a timely manner, and the contents should be carefully checked for omissions or errors to ensure the validity of the questionnaires. After all questionnaires were confirmed to be correct, two independent research assistants entered the survey data in the SPSS system for statistics and analysis.

### Statistical analysis

Statistical analysis was performed using SPSS 25.0 sofware (SPSS Chicago, IL, USA). Categorical variables were presented by frequencies and percentages, while continuous variables were presented by mean and standard deviation. The influence factors were analyzed by ANOVA analysis, Pearson correlation analysis, and linear regression analysis with a test level of α = 0.05. The residuals from the models were tested using the K-S test and evaluated in combination with the histograms and Q-Q plots, indicating an approximately normal distribution.

## Results

### Baseline characteristics and occupational protective behaviors of new nurses

A total of 150 newly recruited nurses were included in the study. Their average age ranged from 19 to 24 (21.55 ± 1.25) years. Among them, there were 18 male nurses (12.0%) and 132 female nurses (88.0%) (Table 1). The total occupational protective behavior score of these nurses was (18.94 ± 3.59). The average score of each item was 1.35 ± 0.26, which was between “partially implemented” and “fully implemented”.

**Table 1:** Influencing factors of occupational protective behaviors

Variables	Group	N	Mean ± SD	F	*p*
**Gender**	Male	18	17.11 ± 3.91	5.461	0.021
	Female	132	19.19 ± 3.49		
**Education**	Junior college	106	19.23 ± 3.94	2.318	0.130
	Bachelor degree or above	44	18.25 ± 2.46		
**Marital status**	Unmarried	123	18.97 ± 3.68	0.040	0.842
	Married	27	18.81 ± 3.22		
**Previous participation in nursing skill-based competitions**	Yes	20	21.05 ± 2.98	8.356	0.004
	No	130	18.62 ± 3.58		
**Standardized training before recruit**	Yes	127	19.20 ± 3.73	4.330	0.039
	No	23	17.52 ± 2.27		
**Experience of needlestick injuries before recruit**	Yes	37	20.51 ± 3.42	9.994	0.002
	No	113	18.42 ± 3.51		

Note: SD: Standard deviation

### Analysis of influencing factors of occupational protective behaviors

The results of ANOVA showed statistically significant (*p* < 0.05) differences between groups in occupational protective behaviors of newly recruited nurses in terms of gender, previous participation in nursing skill-based competitions, standardized training before recruit, and experience of needlestick injuries before recruit (Table 1).

### Correlation analysis of occupational protective behaviors

The work attitude score among newly recruited nurses was 20.24 ± 4.09. Further correlation analysis between work attitude score and occupational protection behaviors showed that there was a significant negative correlation between them (r = -0.324, *p* < 0.001). In addition, the correlation analysis of age, average daily sleep time and occupational protective behaviors showed that there was no correlation between age and occupational protective behaviors (r = 0.158, *p* = 0.053), and a significant positive correlation between average daily sleep time and occupational protective behaviors (r = 0.237, *p* = 0.004) (Table 2).

**Table 2:** Correlation analysis of occupational protective behaviors

	Work attitude score	Age	Average daily sleep time
**Occupational protective behaviors r(** ***p*** **)** ^**a**^	−0.324 (< 0.001)	0.158 (= 0.053)	0.237 (= 0.004)

Note: ^a^r Pearson Correlation Coefficient, *p p*-value (Correlation is significant at the 0.05 level)

### Multifactor analysis of occupational protective behaviors

Multiple linear regression analysis was conducted with occupational protective behavior score as the dependent variable, gender, previous participation in nursing skill-based competitions, standardized training before recruit, experience of needlestick injuries before recruit, work attitude score and average daily sleep time as the independent variables. The results showed that there were statistically significant differences in occupational protective behavior score between different genders, previous participation in nursing skill-based competitions, standardized training before recruit, experience of needlestick injuries before recruit, total score of work attitude and average daily sleep time (*p* < 0.05) (Table 3).

**Table 3:** Multiple linear regression analysis of occupational protective behaviors

	B	SE	β	t	*p*
**Constant**	15.507	2.834		5.472	0.000
**Work attitude score**	−0.267	0.063	−0.304	−4.268	0.000
**Experience of needlestick injuries before recruit**	1.986	0.593	0.239	3.346	0.001
**Previous participation in nursing skill-based competitions**	2.155	0.752	0.205	2.866	0.005
**Average daily sleep time**	0.700	0.279	0.179	2.508	0.013
**Gender**	1.639	0.786	0.149	2.086	0.039

Note: R^2^ = 0.276, adjusted R^2^ = 0.251, F = 10.997, *p* < 0.001

B: Non-standardized coefficient; SE: Standard error; β: Standardization coefficient; R: Coefficient of determination

## Discussion

### Current situation of occupational protective behaviors

Against the backdrop of the considerable health and economic burden of occupational exposures such as needlestick and sharps injuries [3, 4, 7, 8, 17], there has been an increasing emphasis and effort to take measures to minimize the incidence of injuries in recent years. Nevertheless, exposures of HCW to potentially infectious body material occur. A single-center retrospective study with a large sample size found nearly 16% of cases of nonpreventable occupational exposures [1], which was similar to another study [18]. The incidence of occupational exposures such as needlestick injuries among HCW in this study was 13.3%, indicating that the current status of occupational exposures among HCW still cannot be ignored. As one of the main forces of medical activities, the occupational protective behaviors of newly recruited nurses have a direct impact on the quality and safety of nursing care, so an in-depth understanding of the occupational protective behaviors of this group can help to develop appropriate measures to reduce the risk of occupational exposures and improve medical safety.

The results of this study showed that the average score of each item of the occupational protective behavior scale for new nurses was 1.35 ± 0.26, which was between of “partially implemented” and “fully implemented”, with a preference for “partially implemented”. This suggested that the overall awareness of occupational protection of new nurses is relatively weak and needs to be further improved. Meanwhile, it also reflected that although the vast majority of nurses have certain knowledge of occupational protection, they do not pay enough attention to it and do not fully implement it in practical work. A national online survey found a similar conclusion that nursing staff, although attaching importance to hand hygiene and glove use, have poor compliance, especially in emergency situations [19]. Therefore, regular investigation of occupational protective behaviors should be conducted for newly recruited nurses, so as to conduct targeted education of protection behaviors for individuals. Training interventions on standard precautions may be an effective way to reduce the occurrence of occupational exposures [20].

### Correlation analysis of occupational protective behaviors

The total score of work attitude among newly recruited nurses was high (20.24 ± 4.09), indicating that work adaptation of the new nurses was generally poor. At the same time, there was a significant negative correlation between work attitude score and occupational protective behaviors (r = -0.324, *p* < 0.001), indicating that the worse the work adaptability and motivation of newly recruited nurses, the weaker their ability of occupational protective behaviors. It is not difficult to understand that there is an identity shift for newly recruited nurses as they transition from being a student to a competent nurse. There is an imbalance between their academic knowledge and actual clinical experience [21, 22]. Because medical practice involves a degree of apprenticeship, many nurses are prematurely exposed to potentially dangerous sharp tools and objects (e.g., setting up tubes, venipuncture [21], and taking blood samples). They have a gradual process of adjustment in terms of identity and practice. Therefore, it is particularly necessary to timely understand the work adaptation level of newly recruited nurses. For some nurses with poor work adaptability, temporary measures, such as post transfer and “bringing the old with the new”, should be adopted to help them transition to the adaptation period, so as to minimize the incidence of occupational exposures. It is worth noting that the work attitude involved in this study not only covers the adaptation difficulties of the work itself, but also includes factors such as personal negative emotions, poor relationship with colleagues, negative evaluation of leaders, and personality conflicts, which should also be taken into consideration when formulating corresponding intervention strategies. Thus, it can promote the improvement of occupational protective ability of the group more effectively.

### Other influencing factors of occupational protective behaviors

The experience of needle-stick injuries before recruit had an impact on occupational protective behaviors (t = 3.346, *p* < 0.001). That may be related to the fact that nurses with experience of needle-stick injuries have an in-depth understanding of the consequences of inadequate occupational protection, so they pay more attention to the protection of occupational exposures. Therefore, for those who have no previous experience of needle-stick injuries, the risk awareness of this group should be strengthened. Through the form of scenario simulation, the previous experience of other nurses can be collected and reproduced, so that inexperienced nurses can obtain more intuitive and real experience, so as to establish a more profound and comprehensive understanding. In addition, intensive training can also be conducted by means of micro-course of sensory education, so as to promote the attention of nurses without experience of needle-stick injuries to occupational protection.

Previous participation in nursing skill-based competitions had an effect on occupational protection behaviors (t = 2.866, *p* < 0.001). It indicated that nurses who have been trained in nursing skills are more alert to occupational protection, and their standard requirements for nursing operations are more fully internalized, which may be the main reason for their higher level of occupational protective behaviors. Therefore, new nurses should be encouraged to seize the opportunity to actively participate in the nursing skill-based competitions of hospitals and organizations outside the hospital.

The average daily sleep time was positively correlated with occupational protective behaviors (r = 0.237, *p* = 0.004). This indicated that the length of sleep will have a certain impact on the performance of occupational protective behaviors. Although clinical nurses realize the necessity of occupational protection, they tend to ignore the requirements and details of occupational protection due to lack of sleep and varying degrees of fatigue. Occupational exposures are associated with inattention and carelessness [1, 13]. Therefore, it is important to ensure adequate sleep and improve the working environment to avoid inattention caused by adverse factors. For example, invasive procedures are carried out in a light-sensitive and quiet environment [23, 24].

Gender had an effect on occupational protective behaviors (t = 2.086, *p* < 0.05), suggesting that male nurses had worse awareness of occupational protection than female nurses. The reasons may be related to female’s advantages in acquiring occupational protective knowledge and male’s lack of attention to details. Therefore, the training of male nurses on nosocomial knowledge should be strengthened. Daily monitoring and sampling should be conducted to reduce the incidence of their occupational exposures. Another study found that stress and overstrain were one of the main causes of occupational exposures [1]. This phenomenon suggests that mental disorders can increase the occurrence of occupational exposures, which may be associated with reduced adaptability and work efficiency [25]. Therefore, treating diseases that may cause mental disorders, such as coronavirus infection [25], can help to improve work efficiency and avoid the occurrence of occupational exposures due to distraction or inattention.

There were several limitations to the study that should be mentioned. Firstly, the data was collected from a single center. Secondly, some other baseline characteristics that might influence nurses’ occupational protective behaviors were not investigated in the study. Thirdly, the occupational protective behavior scale was self-designed and may not be applicable to other countries or medical units. Therefore, further studies can expand the coverage and diversity of samples to verify the validity and reliability of the scale.

## Conclusion

The overall occupational protective awareness of new nurses is relatively weak and needs to be further improved. By enhancing the adaptability and motivation of new nurses, it may have a positive impact on their ability to improve their occupational protective behaviors. Encouraging new nurses to improve their sleep and actively participate in nursing skill-based competitions is also an important way to improve their occupational protective ability. Guidance and education on occupational protection should be strengthened for high-risk groups with no previous experience of needlestick injuries and male nurses. In addition, this study only explained 25.1% of the total variation of the regression equation, and more relevant influencing factors need to be further explored to provide scientific reference for promoting the soundness of occupational protective awareness and adequate performance of protective behaviors among newly recruited nurses. Meanwhile, a comprehensive multi-center survey can be conducted in future in-depth studies to make the results of this study better generalizable.

### Electronic supplementary material

Below is the link to the electronic supplementary material.

Supplementary Material 1

Supplementary Material 2

Supplementary Material 3

## Data Availability

The datasets generated during the current study are not publicly available due to the limitations of hospital regulations but are available from the corresponding author on reasonable request.

## Abbreviations

HCW: Health care workers
HBV: Hepatitis B virus
HCV: Hepatitis C virus
HIV: Human immunodeficiency virus
Wa: Work attitude scale
SD: Standard deviation
SE: Standard error
