# Supplementary material for: Occupational protection behavior and its influencing factors of newly recruited nurses
Source: BMC Med Educ. 2023 Oct 25;23:797. doi: 10.1186/s12909-023-04780-6 (PMC10601324; doi:10.1186/s12909-023-04780-6)
Supplement: Supplementary file 1 — Supplementary Material 1 [file 12909_2023_4780_MOESM1_ESM.docx]

| Items | Yes | No |
| --- | --- | --- |
| I always work in a very stressful situation. |  |  |
| I am convinced that life is unfair to me. |  |  |
| I find it difficult to concentrate on one task. |  |  |
| If someone had not opposed me, I would have achieved much more. |  |  |
| There are times when I would rather sit and dream than do anything. |  |  |
| There were days, weeks, and months when I didn't want to do anything because I couldn't get up. |  |  |
| Sometimes I have to take orders from people who are not as good as me. |  |  |
| Now, I find it easy to give up on myself. |  |  |
| Some people are so bossy that I fight them even when I know they're right. |  |  |
| I often feel I must stand up for what I think is right. |  |  |
| I don't care what people think of me. |  |  |
| My parents often disapprove of the people I associate with. |  |  |
| I do not blame anyone for wanting to take all that is available in the world. |  |  |
| No matter what I do, I find it hard to get started. |  |  |
| I have a lot of energy sometimes. |  |  |
| Most of the time, life is a struggle for me. |  |  |
| I don't like people around me. |  |  |
| If someone had not opposed me, I would have achieved much more. |  |  |
| I can't concentrate on one thing. |  |  |
| I think twice before I do even trivial things. |  |  |
| I often meet so-called experts who are no better than me. |  |  |
| When things don't go well, I want to quit immediately. |  |  |
| When faced with difficulties or dangers, I always back down. |  |  |
| When I want to correct others' mistakes and help them, my good intentions are often misunderstood. |  |  |
| I usually like working with women. |  |  |
| My plans always seem so difficult that I have to give them up one by one. |  |  |
| I often encounter immediate bosses who take credit for their mistakes and blame them on their subordinates. |  |  |
| My future seemed hopeless. |  |  |
| The future is fickle and it is difficult for one to make serious arrangements. |  |  |
| When I wake up in the morning, I mostly feel well-slept and alert. |  |  |
| My ability to work (study) now is about the same as before. |  |  |
| I always thought life was worth living. |  |  |
| I like to research and read about things related to my current job. |  |  |
| I like many different kinds of games and entertainment. |  |  |
| In everything I do, I expect to succeed. |  |  |
| In my daily life, I am full of things that interest me. |  |  |
| I'm usually calm and not easily agitated. |  |  |

*Work Attitude Scale (Wa)*
