# Supplementary material for: Occupational protection behavior and its influencing factors of newly recruited nurses
Source: BMC Med Educ. 2023 Oct 25;23:797. doi: 10.1186/s12909-023-04780-6 (PMC10601324; doi:10.1186/s12909-023-04780-6)
Supplement: Supplementary file 2 — Supplementary Material 2 [file 12909_2023_4780_MOESM2_ESM.docx]

*Occupational protective behavior scale*

| Item | Judgement | | |
| --- | --- | --- | --- |
|  | Fully implemented | Partially implemented | Not implemented |
| 1. Wear gloves when in contact with blood, body fluids or exposed wounds |  |  |  |
| 2. Wear a mask and gloves during invasive operations such as intravenous intubation, tracheal intubation, thoracentesis |  |  |  |
| 3. Wear a mask and gloves when contacting patients with unknown diagnosis |  |  |  |
| 4. Wear an eye mask, a mask, gloves, a face shield and, if necessary, an isolation gown when in contact with blood, body fluids and rinsing fluids that may be spilled on the patients |  |  |  |
| 5. Wear a mask and gloves during debridement and suture, and wear protective shoes and an eye mask when a lot of washing is needed |  |  |  |
| 6. Debridement and disinfection should be completed after extrusion and bleeding after sharp instrument puncture |  |  |  |
| 7. Wash skin and mucous membranes immediately as soon as they are contaminated with patient blood or body fluids |  |  |  |
| 8. Medical objects contaminated with patient blood or body fluids are wiped or cleaned with disinfectant solution |  |  |  |
| 9. Medical waste shall be classified and disposed of in strict accordance with requirements |  |  |  |
| 10. Wash hands with soap or disinfectant after touching patients or dirt |  |  |  |
| 11. Wear a mask and gloves when preparing and using chemotherapy drugs and chemical disinfectants |  |  |  |
| 12. Wash skin and mucous membranes immediately as soon as they are stained with chemical agents |  |  |  |
